# 8 ^th^ International Meeting on Aortic Diseases

**DOI:** 10.1055/s-0044-1787743

**Published:** 2024-06-11

**Authors:** John A. Elefteriades, Bulat A. Ziganshin

**Affiliations:** 1 AORTA Journal Editorial Office, Aortic Institute at Yale New Haven Hospital, Yale University School of Medicine, New Haven, Connecticut

We are pleased to announce the 8th edition of the International Meeting on Aortic Diseases (IMAD 8) to be organized in Liège in 2024. The meeting will focus on basic research, genetics as well as on the treatment of aortic valves, aneurysms, dissections (types A and B), aortitis and occlusive diseases. **The unedited abstracts from the 8th International Meeting on Aortic Disease are reproduced herein, as supplied by the organizers.** IMPORTANT NOTE: AORTA is pleased to receive full manuscripts corresponding to these abstracts for expedited review and publication.

